# LncRNA HOTAIR promotes proliferation and inhibits apoptosis by sponging miR-214-3p in HPV16 positive cervical cancer cells

**DOI:** 10.1186/s12935-021-02103-7

**Published:** 2021-07-28

**Authors:** Yu Zhou, Yuqing Wang, Mingying Lin, Daiqian Wu, Min Zhao

**Affiliations:** 1 School of Basic Medical Sciences, Wuhan University, Wuhan, Hubei 430071 People’s Republic of China; 2 Department of Cardiology, Zhongnan Hospital of Wuhan University, Wuhan, Hubei 430071 People’s Republic of China; 3 Demonstration Center for Experimental Basic Medicine Education, Wuhan University, Wuhan, Hubei 430071 People’s Republic of China

**Keywords:** Cervical cancer, HPV16 E7, HOTAIR, miR-214-3p, ceRNA

## Abstract

**Background:**

Cervical cancer (CC) is one of the most common gynaecological malignancies all around the world. The mechanisms of cervical carcinoma formation remain under close scrutiny. The long non-coding RNAs (lncRNA) and microRNAs (miRNAs) play important roles in controlling gene expression and promoting the development and progression of cervical cancer by acting as competitive endogenous RNA (ceRNA). However, the roles of lncRNA associated with ceRNAs in cervical carcinogenesis remains unknown. In this study, the expression of long non-coding RNA HOTAIR was investigated in HPV16 positive cervical cancer cells, the candidate miRNAs and target genes were identified to clarify putative ceRNAs of HOTAIR/miRNA in cervical cancer cells.

**Methods:**

The proliferation ability of cells was measured by CCK8 and EdU incorporation assays and cell apoptosis was analyzed by flow cytometry. The expression of HOTAIR, miR-214-3p, HPV16 E7 mRNA were detected by qRT-PCR. As for searching for the interaction between miR-214-3p and HOTAIR, the binding sites for miR-214-3p on HOTAIR was predicted by starbase v2.0 database, then dual-luciferase assay was used to verify the binding sites. In addition, Gene Ontology (GO) and protein–protein interaction (PPI) network analysis of target genes of miR-214-3p were performed with bioinformatics analysis. The potential signal pathway regulated by HOTAIR/miR-214-3p was predicted by KEGG enrichment analysis and confirmed by qPCR and WB analysis in cervical cancer cells.

**Results:**

Our results showed that expression of HOTAIR was up-regulated, while that of miR-214-3p was down-regulated in HPV16-positive cervical cancer cells. The expression status of HPV16 E7 played an important role in regulating expression of HOTAIR or miR-214-3p in cervical cancer cells. HOTAIR knockdown could significantly inhibited cell proliferate ability and promote cellular apoptosis, whereas the inhibition of miR-214-3p expression partially reversed such results. Bioinformatics analysis identified 1451 genes as target genes of miR-214-3p. The Gene ontology (GO) and KEGG Pathway enrichment analysis showed that these target genes were mainly related to regulation of cell communication, protein binding, enzyme binding and transferase activity, and Wnt ligand biogenesis. Pathway enrichment analysis results showed that the predicted target genes were significantly enriched in Wnt/β-catenin signaling pathway. Finally, our results confirmed that miR-214-3p could significantly inhibit β-catenin expression in HPV16 positive cancer cells by qPCR and WB analysis.

**Conclusion:**

HOTAIR could act as a ceRNA through binding to miR-214-3p, promote cell proliferation and inhibit the apoptosis of HPV16 positive cervical cancer. HOTAIR/miR-214-3p/Wnt/β-catenin signal pathway might played important regulated roles in HPV16 positive cervical cancer. Our results provided new insight into defining novel biomarkers for cervical cancer.

## Introduction

Cervical cancer is one of the most common gynecological malignancies in worldwide, with an estimated 604,100 new cases and 341,831 deaths worldwide each year, more than any other gynecologic tumor [1]. HPV (papillomavirus, HPV) infection is the main risk factor for cervical cancer, and 95% of cases are caused by persistent infections with high-risk HPV (hr-HPV) [2]. As is known to all, the integration of hr-HPV DNA into the host cell genome resulting in persistent overexpression of HPV E6 and E7 oncoproteins, subsequently induce immobilization of cells through their inhibitory effects on the tumor suppressor proteins p53 and pRb, respectively [3, 4]. In the recent years, the morbidity and mortality of cervical cancer have been significantly reduced owing to the availability of effective prophylactic vaccines against the most important carcinogenic HPV types including Gardasil and Cervarix [5]. However, although early cervical cancer could be treated with surgery or radiation, the prognosis of advanced cervical cancer patients generally remains poor, and the metastatic cervical cancer was incurable yet and the overall 5 years’ survival rate is only approximately 40% after conventional treatments are used [6]. In addition, the steps and mechanisms of the progression from preneoplastic lesions, cervical intraepithelial neoplasia (CIN) to carcinoma remain unknown. Therefore, the further exploring the mechanism underlying and new therapeutic approaches were urgently required.

Although the persistent infections with hr-HPV was the main risk factor for cervical cancer, most HPV infections could be cleared spontaneously in immuno-competent subjects, so only a minority of women infected with hr-HPV will ultimately develop cervical cancer [7]. Therefore, additional genetic event must have contributed to the initiation and ultimate progression of the cervical cancer. In the recent years, the omics based studies have revealed novel molecular mechanisms leading to cervical carcinogenesis. Epigenetic modifications, including deregulation of microRNA (miRNA), long non-protein coding RNA (lncRNA) and circular RNA (circRNA), have shown to play important roles in cell transformation during distinct stages of cervical intraepithelial neoplasia and cervical carcinoma development [8, 9]. LncRNAs are regulatory transcripts longer than 200 nucleotides, including a 5′ 7-methylguanosine cap and a 3′ poly (A) tail similarly to messenger RNAs [10]. The function of lncRNA is mainly through *cis* and *trans* mode to regulate the gene transcription and post transcriptional modification of target genes [11, 12]. It is generally thought that lncRNAs regulated downstream target genes through competitive endogenous RNA (ceRNA) mechanisms. This means that lncRNA could regulate the expression of gene through competing binding to miRNAs at the post-transcriptional level. For example, lncRNAs regulated target genes transcription by interacting with chromatin-modifying complexes at specific regulatory regions, or as a molecular sponge of transcription factors and miRNAs [13]. Numerous experimental results confirm that lncRNAs could play a crucial role in the development of cervical cancer [8, 14]. Around 14 lncRNAs have shown to be altered in cervical carcinoma affecting important metabolic pathways such as PI3K/AKT, Notch signaling and MAPK pathway [14]. Moreover, some lncRNAs, including MALAT1, CCEPR, and TMPOP2, could interact with HPV16 E6 and E7 oncogene to enhance its carcinogenesis effect in the development of cervical neoplasia [15, 16].

The lncRNA HOTAIR (HOX transcript antisense intergenic RNA) was the first *trans* regulatory long non-coding RNA to be found, encoded by the antisense strand of the HOXC gene located in the chromosome 12 q13.13 [17]. The HOTAIR 5′ domain could bind the zeste homolog 2 (EZH2) and to silence the target gene nemo-like kinase (NLK) [18]. Recently, HOTAIR has been conformed as molecular sponge for several miRNAs in various malignant tumors including colorectal cancer, hepatocellular carcinoma, pancreatic carcinoma and cervical cancer [19–21]. However, the precise role and function of HOTAIR in cervical cancer remained unclear, only a relatively small number of studies have associated HOTAIR with cervical cancer. The current research showed that HOTAIR was highly expressed and could be used as an indicator of poor prognosis of cervical cancer [22, 23]. A recent study has revealed that HPV16 E7 inhibited the recruitment of the PRC2 complex and promoted the development of cervical cancer by competing the binding site of the PRC2 complex on HOTAIR [24]. HOTAIR could regulate the miR-143-3p/BCL2 axis and miR-20a-5p/HMGA2 axis contributing to cell proliferation and metastasis [25, 26]. These researches indicated that HOTAIR might play an important roles in cervical cancer. However, the relevant research results were still very limited in cervical cancer.

Numerous studies have analyzed the miRNAs expression to identify significant variations during the transition from low to high grade cervical neoplasia and to invasive cervical cancer [27, 28]. Many miRNAs (let-7b, let-7c, miR-21, miR-23b, miR-196b, miR-143, miR-126-3p, miR-20b-5p, miR-451a, and miR-144-3p) were identified as potential biomarkers for cervical cancer diagnosis, prognosis and cancer stage, even have been shown effective for diagnosis of cervical adenocarcinoma and high grade intraepithelial lesions [29, 30]. MiR-214-3p was a miRNA involved in many cancer, including lung cancer, breast cancer, colon cancer, endometrial carcinoma, and pancreatic cancer in humans [31, 32]. In cervical cancer, miR-214-3p has been found play an important roles in regulating cell proliferation, apoptosis, cell invasion, metastasis and angiogenesis [33, 34]. Unfortunately, although the potential effectiveness was identified in cervical cancer, the mechanism and status of miR-214-3p involve in ceRNA network in cervical cancer cells needs to be addressed. Moreover, there was no report about the correlation between lncRNA HOTAIR and miR-214-3p in cervical cancer cells so far. Therefore, more investigations were needed in order to deep research the interaction between lncRNA HOTAIR and miR-214-3p in cervical cancer.

In this study, we detected the expression of HOTAIR and miR-214-3p in cervical cancer cells, conducted the target genes and pathway enrichment analysis with bioinformatics analysis. The effects of HOTAIR-miR-214-3p ceRNA network was specifically investigated to clarify the mechanism and potential target genes of cervical carcinogenesis.

## Materials and methods

### Cell culture and reagents

The human cervical cancer cell lines SiHa, C-33A and immortalized cervical epithelial End1/E6E7 cells (ATCC, Rockville, MD) were all cultured in DMEM (Hyclone) supplemented with 10% fetal bovine serum (FBS, Gibco) and 1% penicillin/streptomycin cells at 37℃ in a humidified 5% CO_2_ atmosphere. All the consumables for cell culture were from JET BIOFIL® (Guangzhou, China). Primary antibodies against eIF4E (C46H6, rabbit mAb, #9742), β-catenin (D10A8, rabbit mAb, #8480), goat peroxidase (HRP)-conjugated secondary antibodies (#7074) and antibody against β-actin (D6A8, rabbit mAb, #8457) were all purchased from Cell Signaling Technology, China.

### RNA oligos and plasmid

All RNA oligos including small interference RNAs (siRNAs), miRNA mimics, negative control (NC) and inhibitor were chemically synthesized by Shanghai GenePharma Company (Shanghai, China). Two siRNAs for HOTAIR was designed: si-HOTAIR-1 and si-HOTAIR-2, the better siRNA would be used in final experiment. In this study, cells were transfected with 160 nM of siRNAs and negative control, and the final used concentration of miR-214-3p and mimics negative control was 80 nM. The following sequences of RNA oligos were used: si-HOTAIR-1 (GAGGCGCUAAUUAAUUGAUTTAUCAAUUAAUUAGCGCCUCTT), si-HOTAIR-2 (GCA CAGAGCAACUCUAUAATTUUAUAGAGUUGCUCUGUGCTT), si-NC (UUCUCCGAACGU GUCACGUTTACGUGACACGUUCGGAGAATT); miR-214-3p mimics (ACAGCAGGCACAG ACAGGCAGUUGCCUGUCUGUGCCUGCUGUUU), mimics NC (UUGUACUACACAAAAG UACUG). The shRNA for HPV16E7 was purchased from Genepharma (Suchow, China). The expression plasmid of HPV16 E7, pLXSN-E7 was preserved in our laboratory. In addition, we constructed the expression plasmid of HOTAIR functional motif, pEGFP-C1-HOTAIR-A, which contained the binding sites of miR-214-3p. The PCR amplification fragment of HOTAIR functional motif was insert between *Mlu*I and *Hind* III sites on the EGFP-C1 vector.

### Dual luciferase assay

For structure of of HOTAIR luciferase reporter plasmid, we firstly predicted the binding sites of miR-214-3p on HOTAIR by bioinformatics analysis (starbase v2.0 database), then the fragment of 3`-UTR of HOTAIR containing miR-214-3p binding sites was amplified by PCR and inserted it into pMIR-REPORTTM Luciferase vector. Cell transfection was performed with Lipofectamine 2000 reagent (Invitrogen, U.S.A.). Then, luciferase reporter plasmid, renilla luciferase plasmid and miR-214-3p mimics/inhibitor were co-transfected into cells. After 24 h transfection, the luciferase activities were measured with a dual luciferase reporter assay system (Promega, Madison, U.S.A.) according to the manufacturer’s instructions. Renilla luciferase intensity was normalized to firefly luciferase intensity.

### RNA isolation and qRT-PCR

Total RNA was extracted with TRIzol reagent (Invitrogen). M-MLV reverse transcriptase (Promega, USA) and OligodT(15) were used to convert RNA into cDNA. Then quantitive RT-PCR was adopted to detect the related expression of HPV16 E7, HOTAIR, miR-214-3p and β-catenin. For miR-214-3p was normalized to control U6 snRNA, the others were normalized to GAPDH. QRT-PCR analysis was performed with SybrGreen qPCR Mastermix (DBI® Bioscience, Germany) following the manufacturer’s protocol. All the primers for PCR were shown in Table 1.

**Table 1:** Sequences of primers

Primers	Sequences
miR-214-RT	5′-GTCGTATCCAGTGCAGGGTCCGAGGTATTCGCACTGGATACGACACTGCC-3′
U6-RT	5′-GTCGTATCCAGTGCAGGGTCCGAGGTATTCGCACTGGATACGACAAAATATG-3′
miR-214-3p-real-F	5′-CGCCGACAGCAGGCACA-3′
U6-real-F	5′-GCGCGTCGTGAAGCGTTC-3′
miR-real-GP	5′-GTGCAGGGTCCGAGGT-3′
HOTAIR-real-F	5′-GGGACAGAAGGAAAGCCCTC-3′
HOTAIR-real-R	5′-TTGAGAGCACCTCCGGGATA-3′
GAPDH-real-F	5′-CTGCACCACCAACTGCTTAG-3′
GAPDH-real-R	5′-TTCTGGGTGGCAGTGATG-3′
Luc-HOTAIR-F	5′-CGACGCGTAGAAGCAAAGGTCCAG-3′
Luc-HOTAIR-R	5′-CGCAAGCTTAAGTGCATACCTACC-3′
HOTAIR-A-F	5′-CGAAGCTTAGAAGCAAAGGTCCAG-3′
HOTAIR-A-R	5′-CGACGCGTAAGTGCATACCTAC-3′

### Cell viability and proliferative capacity

Cells were cultured on a 96-well plate and transfected with siRNAs or mimics for various times. Cell viability was then measured by the CCK-8 kit (Beyotime Biotechnology, Shanghai, China) according to the manufacturer’s instructions. For CCK8 assay, 1000 per well cells were seeded into 96-well plates 24–48 h after transfection and the absorbance were detected by microplate reader at 450 nm. Cell proliferation capacity was evaluated by 5-ethynyl-2'-deoxyuridine (EdU) incorporation assay. EdU is a thymidine analogue that can be inserted into the replicating DNA instead of thymidine (T) during cell proliferation. The fluorescent dye Apollo® binds specifically to EdU which has inserted into DNA and could be detected by excitation light at 488 nm. Green light represents the growing cell. Hoechst stained cell nuclei represent the total number of cells. In EdU incorporation assay, 1 × 10^4^–1.5 × 10^4^ cells were seeded into 96-well plates 24 or 48hplates 24 or 48 h after transfection and the next steps were following the manufacturer’s protocol of EdU Imaging Kit (Ribobio, China), the fluorescent images were acquired by the fluorescence microscope (Olympus, Japan). All the experiments were performed for at least three times.

### Cell apoptosis analysis by flow cytometry

Following the manufacturer’s protocol of the Annexin V-FITC/PI Dual Staining Cell Apoptosis Detection Kit (Bestbio, China), transfected cells were plated in 12-well plates for 36 h, and gathered together by centrifugation after washing with ice-cold PBS. Then the cells were resuspended in Annexin V binding buffer and incubated with FITC-conjugated Annexin V antibody and propidium iodide (1:100 dilutions) for 15 min at room temperature. The fluorescence was detected by Beckman CytoFLEX FCM (USA).

### Western blotting analysis

Cells were harvested and boiled in lysis buffer containing protease inhibitors. Total protein lysates were separated by 10% SDS-PAGE gel and electrophoretically transferred PVDF (polyvinylidene difluoride) membranes (Millipore). Then the PVDF membranes were blocked by 5% skim milk for 3–4 h at room temperature and incubated with primary antibodies (1:1000) at 4℃ overnight, washed with TBST buffer, and incubated again with an appropriate HRP-conjugated secondary antibody (1:5000) at room temperature for 1 h. The enhanced chemiluminescence (ECL) was used to detect the results.

### Bioinformatics analysis

In this study, a integrating bioinformatics analysis was performed to analyze the function and interaction between LncRNA HOTAIR and miR-214-3p. The starbase v2.0 (http://starbase.sysu.edu.cn/) was used to predict the binding sites of HOTAIR and miR-214-3p. In addition, the targeted genes of miR-214-3p were predicted using TargetScan7.2 (http://www.targetscan.org/) and miRWALK3.0 (http://zmf.umm.uni-heidelberg.de/apps/zmf/ mirwalk2) [35]. Subsequently, Gene Ontology (GO) and Kyoto Encyclopedia of Genes and Genomes (KEGG) pathway enrichment analysis were performed using the Search Tool for the Retrieval of Interacting Genes (STRING 11.0; https://string-db.org/) database, and FDR < 0.05 was set as the cut-off criterion.

### Statistical analysis

All statistical analyses were performed using SPSS19 (SPSS Inc., Chicago, IL, USA). The results were expressed as the means ± S.D. The statistical significance of the results was analyzed using two-tailed Student's *t*-test. The difference was considered statistically significant at *p* < 0.05 and indicated by asterisks in the figures. All experiments were repeated independently more than three times.

## Results

### HOTAIR expression is correlated with HPV16E7 in cervical cancer cells

**Fig. 1:**
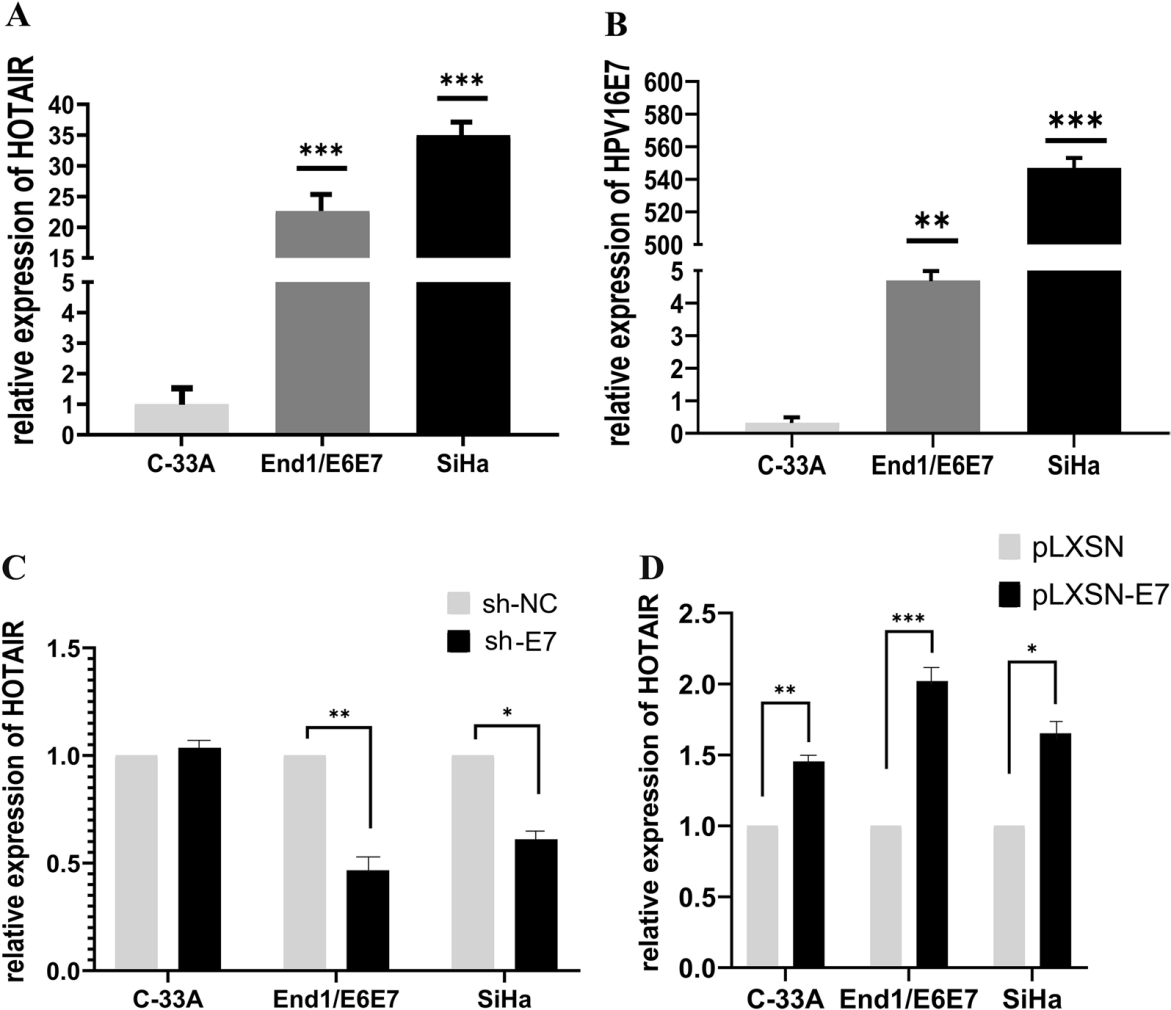
HPV16 E7 promotes HOTAIR expression in cervical cancer cells by qPCR analysis. **A** HOTAIR was highly expressed in End1/E6E7 and SiHa cells, but HOTAIR level was very low in C-33A cells (HPV negative). **B** HPV16 E7 was highly expressed in End1/E6E7 and SiHa cells compared with C-33A cell. **C** Knockdown HPV16 E7 significantly inhibited the expression of HOTAIR in End1/E6E7 and SiHa cells, but there was no alteration in C-33A cell. **D** Upregulated expression of HPV16 E7 significantly promoted HOTAIR expression in all cervical cancer cells. The mean values and standard error were obtained from three independent experiments. GAPDH was used as an endogenous control gene. *P < 0.05, **P < 0.01, ***P < 0.001

In this experiment, we detected the expression level of HPV16E7 and HOTAIR in End1/E6E7, SiHa and C-33A cells. End1/E6E7 cell lines was immortalized cervical epithelial cell where HPV16 E6 and E7 has been stably transfected, and SiHa cell line has integrated with HPV16 genome, and HPV negative C-33A cells was used as negative control to normalize in this study. The results showed that HOTAIR appeared to be at a high expression level in End1/E6E7 and SiHa cells, the expression level of HOTAIR was enhanced by approximately 23 and 35 folds as compared with C-33A cells, respectively (Fig. 1A). Simultaneously, the high expression of HPV16 E7 was detected in End1/E6E7 and SiHa cells, and E7 expression were enhanced by 4.5 and 550 times compared to C33A cells, respectively. Next, we detected the expression level of HOTAIR when HPV16 E7 expression was knocked down or up-expressed in those three cell lines, respectively. Our results showed that after knocked down HPV16 E7 with sh-E7, HOTAIR expression in End1/E6E7 and SiHa cells was significant 55% and 40% reduced compared to control cells, respectively. But there was no obvious change in C33A cells (Fig. 1C). Notably, while we exogenously up-expressed HPV16 E7 by transfected with pLXSN-E7, HOTAIR expression in those three cell lines were all upregulated, the HOTAIR level were increased by approximately 40%, 100% and 70% in C33A, End1/E6E7 and SiHa cells compared to control cells, respectively (Fig. 1D). Our results confirmed that HOTAIR expression have positive correlation with HPV16 E7, suggested that there was indeed interaction between lncRNA HOTAIR and HPV16 E7 in cervical cancer cells.

### HOTAIR plays a growth promoting role in cervical cancer cells

**Fig. 2:**
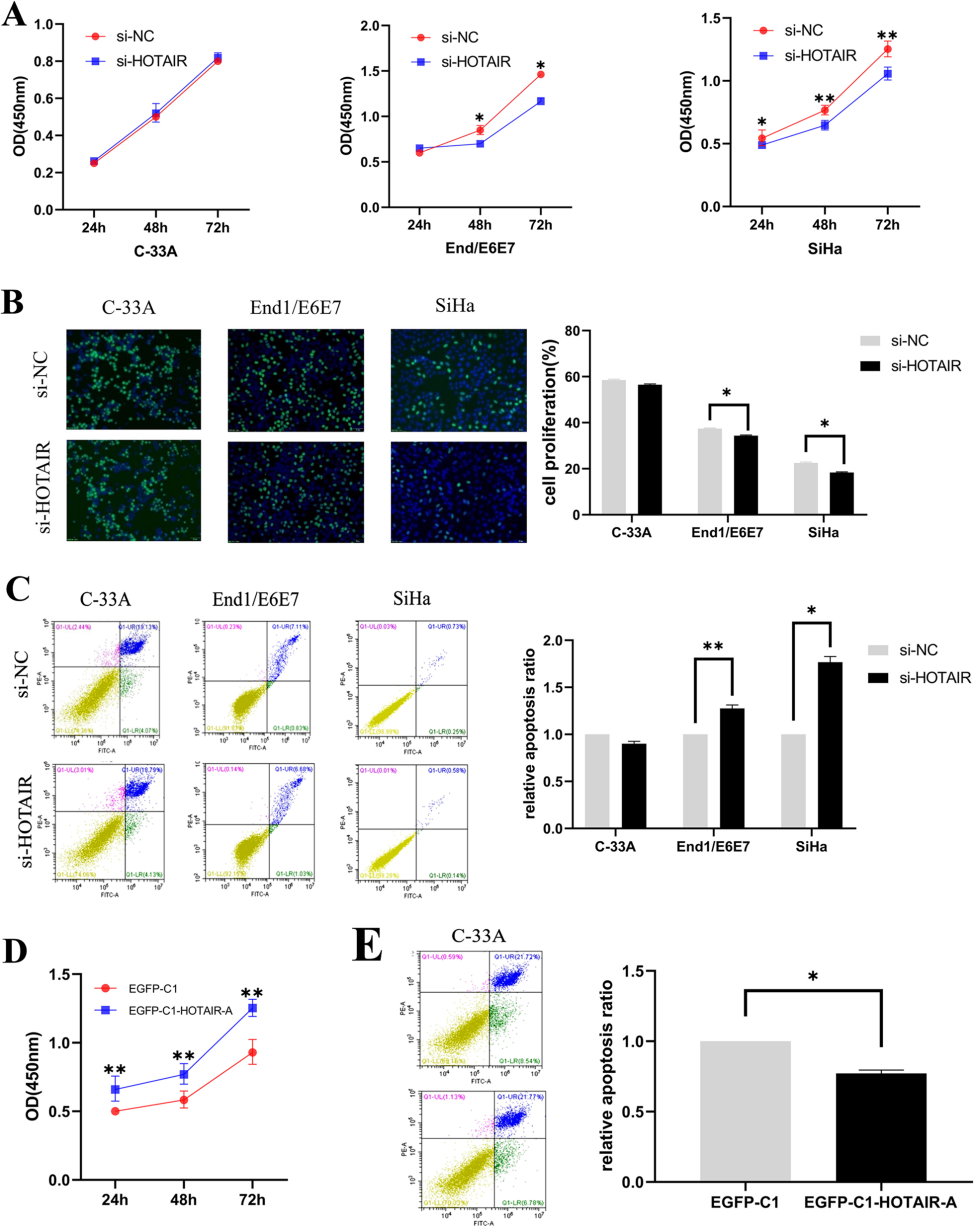
HOTAIR plays a growth promoting role in cervical cancer cells. **A** C-33A, End1/E6E7 and SiHa cells were transfected with si-HOTAIR and si-NC for 24 h, 48 h and 72 h, the cell viability was measured by CCK8 assay. Results showed that the cell viability of End1/E6E7 and SiHa cells were significantly inhibited compared to control cells, but there was no significant change in C-33A. **B** Cell proliferation was measured in C-33A, End1/E6E7 and SiHa cells transfected with si-HOTAIR and si-NC by EdU incorporation assay. The cell proliferation of End1/E6E7 and SiHa cells were significantly inhibited after HOTAIR knocked down. The representative fields were photographed and counted at × 200 magnification. The cells were counted in five different fields per assay under the microscope. **C** The cell apoptosis was analyzed by Flow cytometry analysis. Compared with cells transfected with si-NC, the percentage of apoptotic cells in SiHa and End1/E6E7 were significantly increased after transfected with si-HOTAIR. **D** The cell viability was obvious increasing when overexpressed functional motif of HOTAIR in C-33A cells by CCK8 assay. **E** The early apoptosis of C-33A cell was significantly decreased after overexpressed HOTAIR-A by flow cytometry analysis. The mean values and standard error were obtained from three independent experiments. *P < 0.05, **P < 0.01

To investigate the effect of HOTAIR on the phenotype of cervical cancer cells, the CCK8 and EdU incorporation assays were performed to detect the cell viability and cell proliferation, respectively. In this study, we detected cell viability at 24 h, 48 h and 72 h after HOTAIR siRNA transfection. As shown by CCK8 assay, the cell viability of End1/E6E7 and SiHa cells were significantly inhibited at 48 h and 72 h compared to control cells, respectively (Fig. 2A). Additionally, knockdown of HOTAIR also significantly inhibited the proliferative abilities of End1/E6E7 and SiHa cells. After HOTAIR siRNA transfection 48 h, the cell proliferation capacity of End1/E6E7 and SiHa cells were approximately 12% and 21% reduced compared to control cells, respectively (Fig. 2B). No significant changes were found in C-33A cells by CCK8 and EdU incorporation assay, it was HOTAIR remained a low expression level in C-33A cells. We used FITC/PI double staining assay to detect cell apoptosis by flow cytometry. Results showed that the apoptotic rate were approximately 30% and 70% increased respectively after knocking down HOTAIR in SiHa and End1/E6E7 cells compared to control cells, but the apoptotic rate of C-33A cells remained almost unchanged (Fig. 2C). By contrast, the cell viability showed an obvious increase in C33A cells transfected with HOTAIR functional motif at 24 h, 48 h and 72 h later (Fig. 2D). The apoptotic rate of C-33A cells showed a 25% decrease when HOTAIR functional motif was overexpressed by transfected with pEGFP-C1-HOTAIR-A in C-33A cells (Fig. 2E). Taken together, these results suggested that HOTAIR could promote proliferation and inhibit apoptosis in HPV16 positive cervical cancer cells.

### MiR-214-3p is downregulated expression and acts as a suppressor in HPV16 positive cervical cancer cells

**Fig. 3:**
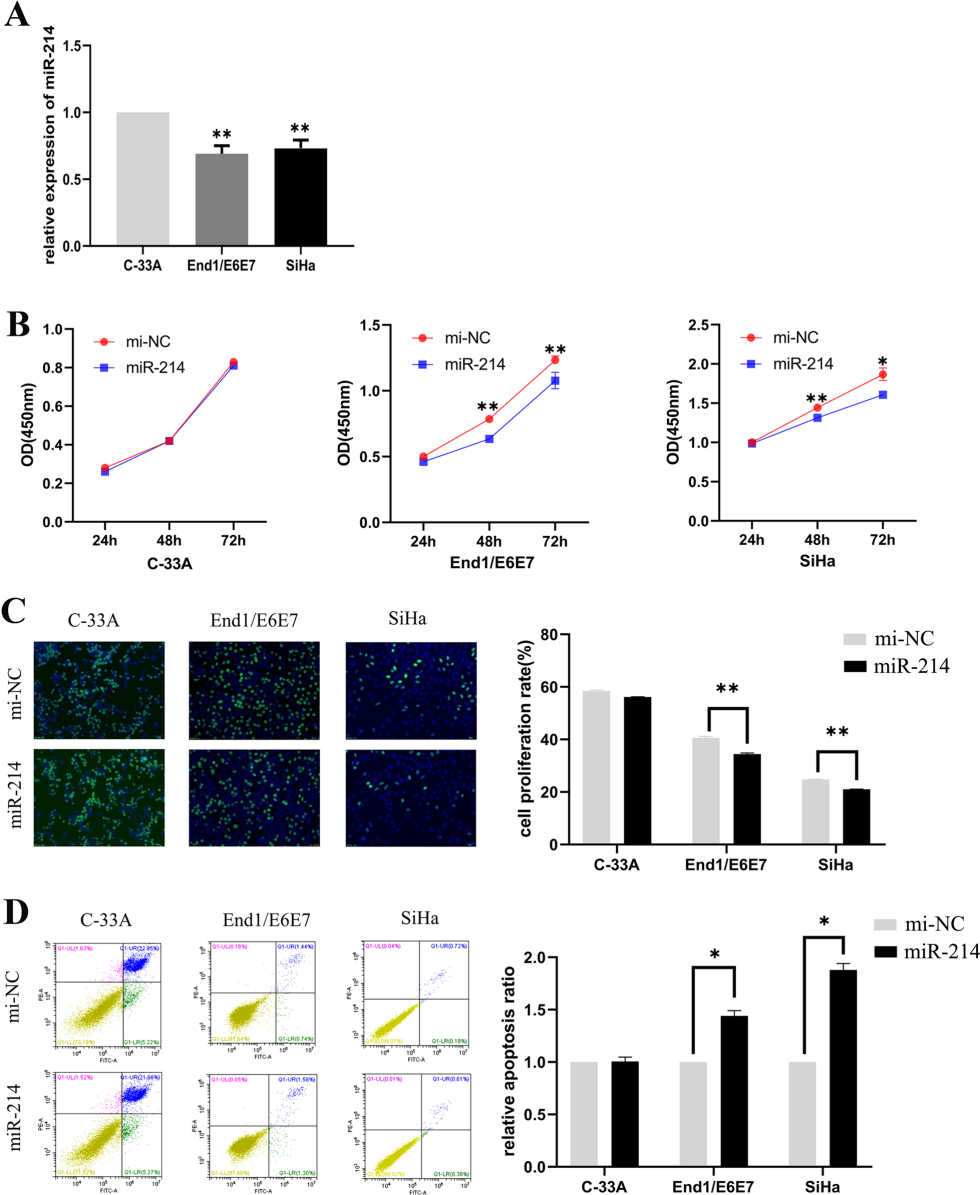
miR-214-3p was down regulated expression in HPV16 positive cells and acted as a suppressor gene in cervical cancer cells. **A** The expression miR-214-3p in End1/E6E7 and SiHa cells was significantly lower than in C-33A cells. **B**, **C** C-33A, End1/E6E7 and SiHa cells were transfected with miR-214-3p mimic and negative control for 24 h, 48 h and 72 h, the cell viability was measured by CCK8 assay and cell proliferation was measured by EdU incorporation assay. Results showed that the cell viability and proliferation of End1/E6E7 and SiHa cells were significantly inhibited, but there was no significant change in C-33A cells. The representative fields were photographed and counted at × 200 magnification. The cells were counted in five different fields per assay under the microscope. **D** Upregulated expression of miR-214-3p increases early apoptotic population in End1/E6E7 and SiHa cervical cancer cells after transfected with miR-214-3p mimic. The mean values and standard error were obtained from three independent experiments. *P < 0.05, **P < 0.01.

As shown in Fig. 3A, the expression level of miR-214-3p was 30% and 25% reduced in End1/E6E7 and SiHa cells comparing to HPV negative C-33A cells, respectively. We also investigated the effect of miR-214-3p on cell proliferation and apoptosis in three cell lines. Results revealed that overexpression of miR-214-3p could inhibit proliferation of End1/E6E7 and SiHa cells, the cell viability were approximately 30% and 20% reduced compared to control cells at 48 h later, respectively (Fig. 3B). Similarly, the experimental results of 72 h were consistent with those of 48 h. In addition, after miR-214-3p transfection 48 h, the cell proliferation capacity of End1/E6E7 and SiHa cells were approximately 18% and 25% reduced compared to mi-NC transfected cells, respectively (Fig. 3C). We also observed that End1/E6E7 and SiHa cells exhibited significant difference in percentage of early apoptosis while miR-214-3p overexpressed (Fig. 3D). However, there was no significant alteration in cell proliferation capacity and early apoptotic cells when miR-214-3p was upregulated in C33A cells. Notability, these results were completely contrary to the function and effect of HOTAIR in cervical cancer cells.

### HOTAIR serves as a sponge for miR-214-3p

**Fig. 4:**
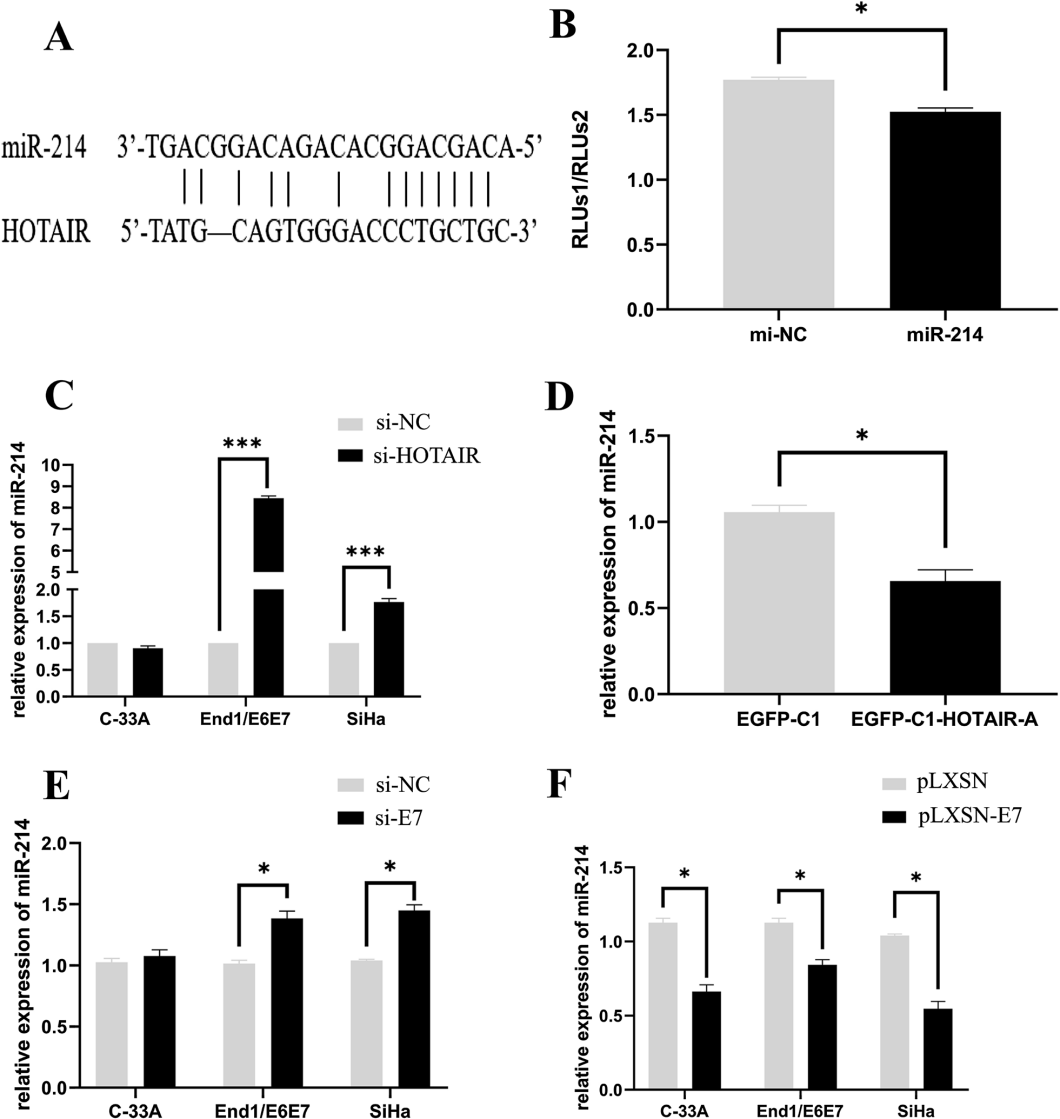
Validation of HOTAIR as a sponge of miR-214-3p in cervical cancer cells. **A** Bioinformatics analysis of miR-214-3p response elements on HOTAIR sequence. The seed sequences of miR-214-3p (CAGCAGG) were fully complementary to the 1807–1813 bp on HOTAIR. **B** The activity of luciferase was significantly reduced after co-transfected with miR-214-3p mimics and HOTAIR reporter plasmid in HEK-293 T cells overexpressing miR-214-3p. **C** Knockdown of HOTAIR significantly upregulated the expression of miR-214-3p in HPV16 positive cells, but not in HPV negative C-33A cells. **D** HOTAIR-A (contain MRE) obviously inhibited miR-214-3p expression in C-33A cells. **E** The expression level of miR-214-3p was significantly upregulated when HPV16 E7 was inhibited in End1/E6E7 and SiHa cells, respectively. **F** The expression level of miR-214-3p was significantly reduced when HPV16 E7 expression was enhanced in C33A, End1/E6E7 and SiHa cells, respectively. The mean values and standard error were obtained from three independent experiments. *P < 0.05, ***P < 0.001

To clarify whether there was an interaction between HOTAIR and miR-214-3p, we performed an integrating bioinformatics analysis to identify the potential binding site on two non-coding RNAs with starbase v2.0 database. Finally, we found that the 7 bp seed sequences (CAGCAGG) at 5′ end of miR-214-3p were fully complementary to the 1807–1813 bp on HOTAIR (Fig. 4A). We performed dual luciferase assay to verify the function of this binding site as a microRNA response elements (MREs)on HOTAIR. First, we obtained a 600 bp fragments of the predicted binding site flank sequence (including the upstream 300 bp and downstream 300 bp) by PCR. Then, this 600 bp fragment was inserted into a reporter plasmid which expresses luciferase, the renilla luciferase plasmid was used as a control. In order to avoid the influence of abnormal oncogene expression patterns and HPV interference in tumor cells, we performed the dual luciferase assay in HEK-293 T cells. The results showed that the activity of luciferase was approximately 20% reduced after co-transfected with miR-214-3p mimics and HOTAIR reporter plasmid compared to control cells (*P* < 0.01), indicating that this site was indeed a response element on HOTAIR sequence (Fig. 4B). HOTAIR could rely on this response element to serve as a sponge against miR-214-3p, and inhibited the function of miR-214-3p in cervical cancer cells.

Next, we focused our analysis on the expression of miR-214-3p in cells transfected with HOTAIR siRNA. Results suggested that the expression of miR-214-3p was significantly enhanced by approximately 8.5 and 1.8 folds in HPV16 positive End1/E6E7 and SiHa cells after HOTAIR knocked down, respectively (Fig. 4C). However, in HPV negative C-33A cells, knockdown of HOTAIR did not significantly change miR-214-3p expression. Conversely, as shown in Fig. 4D, it was found that miR-214-3p expression was obviously reduced by 40% when HOTAIR-A plasmid which contained the binding sites of miR-214-3p was transfected into C-33A cells.

To verify the correlation between HPV16 E7 and HOTAIR expression, we detected the alteration of miR-214-3p expression when knocked down and overexpressed HPV16 E7, respectively. Results indicated that miR-214-3p expression was significantly enhanced by 40–50% in End1/E6E7 and SiHa cells transfected with sh-E7, respectively (Fig. 4E). Conversely, miR-214-3p expression was significantly 30–50% reduced in End1/E6E7 and SiHa cells when HPV16E7 was upregulated in these cells by transfected with E7 expression plasmid into cells. Particularly, miR-214-3p expression was decreased by more than 40% in C33A cells by transfected with E7 expression plasmid (Fig. 4F). Considering all the results above, it suggested that HOTAIR could act as a molecular sponge of miR-214-3p, to inhibit the action of miR-214-3p in HPV16 positive End1/E6E7 and SiHa cells. Notably, HPV16 E7 maybe play a key regulatory role in this process, because HOTAIR inhibition roles against miR-214-3p no longer existed due to the loss or decline of E7 expression in cervical cancer cells.

### HOTAIR controls β-catenin expression via miR-214-3p

**Fig. 5:**
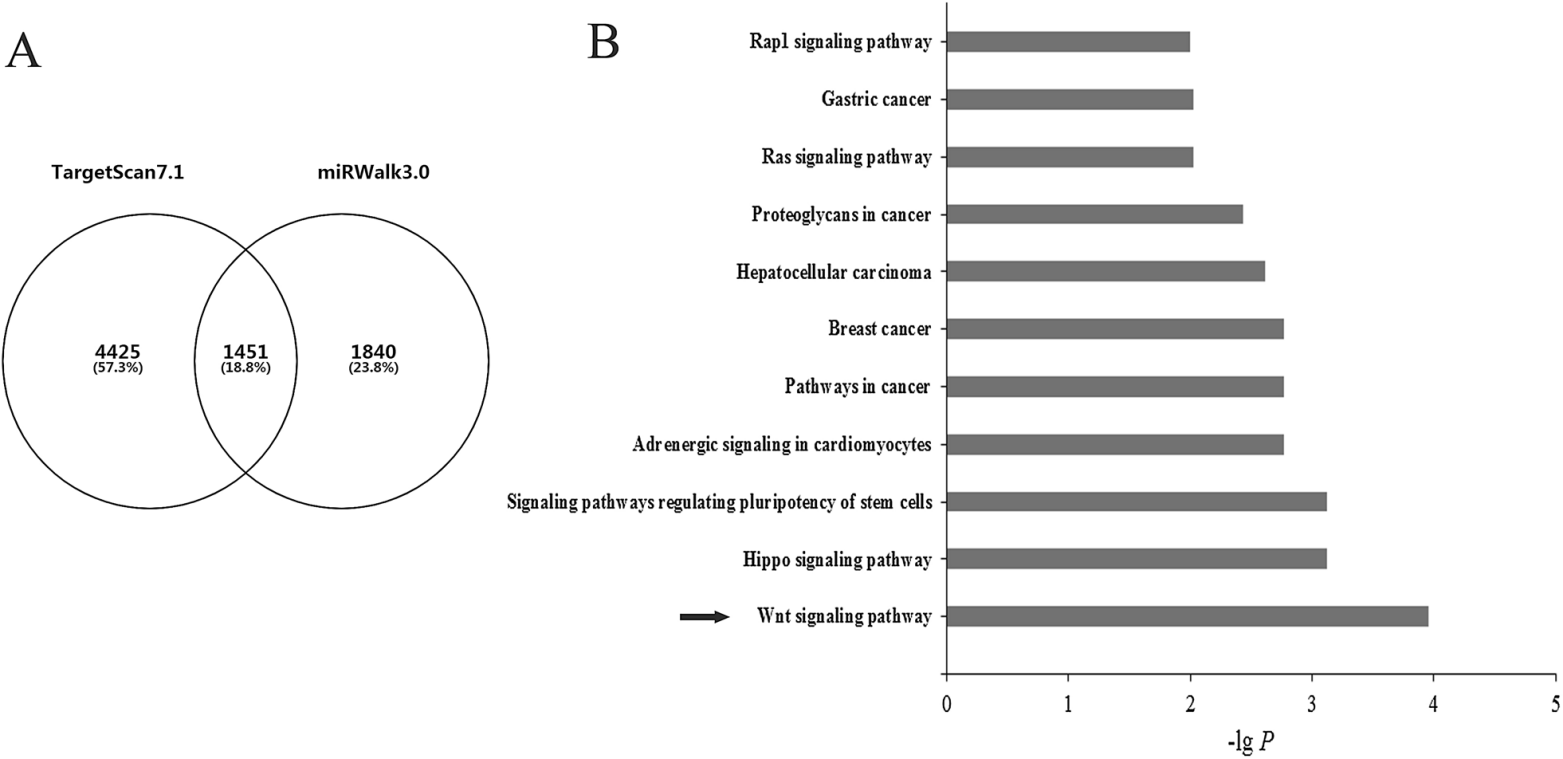
The signal pathway of miR-214-3p target genes was predicted with bioinformatics analysis. **A** The target genes was predicted with TargetScan7.1 and miRWALK3.0. Totally1451 genes were screened by two databases analysis. **B** KEGG pathway enrichment analysis was performed to screen the key pathway related with miR-214-3p. The arrow indicates the highest ranking signaling pathway

To explore the potential signaling pathway regulated by miR-214-3p in cervical cancer cells, we performed the bioinformatics analysis of miR-214-3p. Firstly, target genes of miR-214-3p were predicted with TargetScan7.1 and miRWALK3.0. After discarding repeatedly predicted genes, the predicted target genes were further analyzed by performing a negative correlation analysis. Finally, a total of 1451 genes were predicted as target genes of miR-214-3p (Fig. 5A). Next, to initially comprehend the action of the genes, we submitted predicted genes to STRING11.0, to perform the GO and KEGG Pathway enrichment analysis. GO enrichment analysis results showed that mostly enriched in biological processes (BP) terms related to cellular process and regulation of biological process, reactome pathways (RCTM) terms included Wnt ligand biogenesis and trafficking, nuclear Events (kinase and transcription factor activation), MAPK targets/Nuclear events mediated by MAP kinases9 (See Table 2). Pathway enrichment analysis results showed that the predicted target genes were significantly enriched in Wnt signaling pathway, hippo signaling pathway, signaling pathways regulating pluripotency of stem cells, pathways in cancer (Fig. 5B). Taken together, these results suggested that Wnt signal pathway maybe probably key pathway related with miR-214-3p.

**Table 2:** GO and pathway enrichment analysis of target genes of miR-214-3p

Term ID	Term description	Gene count	Strength	FDR
*Biological processes*				
GO:0009987	Cellular process	1186	0.03	1.1E−04
GO:0065007	Biological regulation	979	0.04	1.1E−04
GO:0050789	Regulation of biological process	942	0.05	2.92E−05
GO:0050794	Regulation of cellular process	896	0.05	2.88E−05
GO:0008152	Metabolic process	818	0.05	1.1E−04
*Cell component*				
GO:0005623	Cell	1312	0.03	1.39E−07
GO:0005622	Intracellular	1225	0.06	7.97E−16
GO:0043226	Organelle	1062	0.05	5.67E−09
GO:0043229	Intracellular organelle	1042	0.05	1.28E−08
GO:0005737	Cytoplasm	993	0.07	1.68E−11
*Molecular function*				
GO:0005488	Binding	1006	0.05	3.75E−06
GO:0005515	Protein binding	614	0.09	1.33E−06
GO:0043167	Ion binding	541	0.07	1.1E−03
GO:0003824	Catalytic activity	499	0.07	2.6E−03
GO:0097159	Organic cyclic compound binding	464	0.06	3.41E−02
*KEGG pathways*				
hsa04310	Wnt signaling pathway	32	0.47	1.1E−04
hsa04390	Hippo signaling pathway	30	0.42	7.5E−04
hsa04550	Signaling pathways regulating pluripotency of stem cells	28	0.43	7.5E−04
hsa04261	Adrenergic signaling in cardiomyocytes	27	0.41	3.7E−03
hsa05200	Pathways in cancer	67	0.24	3.7E−03
*Reactome pathways*				
HSA-3238698	WNT ligand biogenesis and trafficking	11	0.75	2.23E−02
HSA-198725	Nuclear events (kinase and transcription factor activation)	9	0.68	4.12E−02
HSA-450282	MAPK targets/ nuclear events mediated by MAP kinases	10	0.63	4.12E−02
HSA-8986944	Transcriptional regulation by MECP2	16	0.56	2.23E−02
HSA-187037	Signaling by NTRK1 (TRKA)	18	0.5	2.23E−02

*GO* gene ontology, *KEGG* Kyoto Encyclopedia of Genes and Genomes, *FDR* false discovery rate

**Fig. 6:**
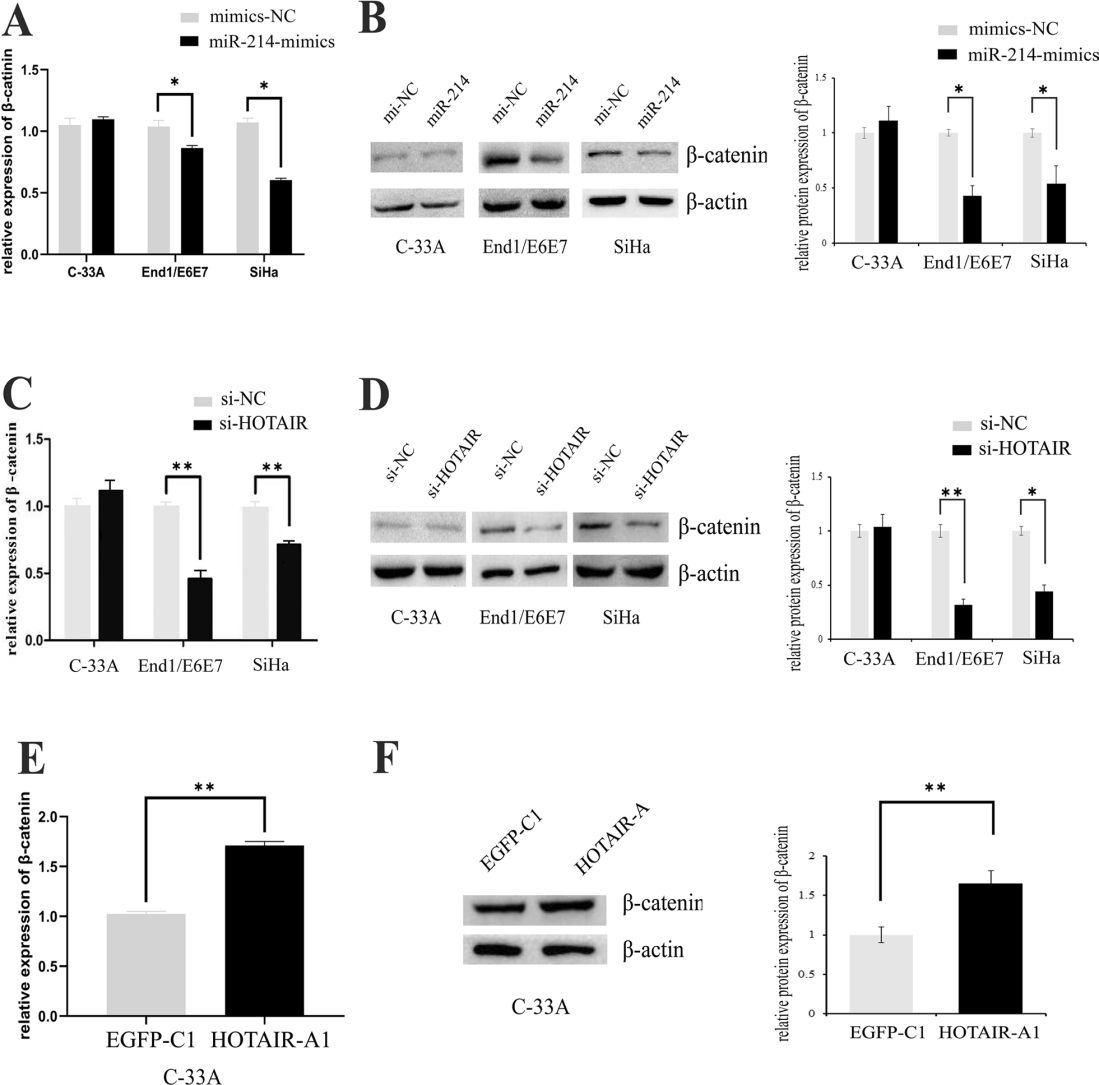
HOTAIR/miR-214–3 axis mediated the expression of β-catenin in HPV16 positive cervical cancer cells. (**A**, **B**) Over-expression of miR-214-3p could inhibit the expression of β-catenin mRNA and protein by qPCR (**A**) and western blotting analysis (**B**) in End1/E6E7 and SiHa cells. **C**, **D** Knocking down HOTAIR could inhibit the expression of β-catenin mRNA and protein by qPCR (**C**) and western blotting analysis (**D**) in End1/E6E7 and SiHa cells. **E**, **F** Overexpressed of HOTAIR-A (contain MRE) obviously up-regulated the expression of β-catenin mRNA (**E**) and protein (**F**) in C-33A cells. *P < 0.05, **P < 0.01

In Wnt signaling pathway, β-catenin has been identified a verified target of miR-214-3p by bioinformatics analysis. In this part we aimed to investigate the effect of HOTAIR/miR-214-3p on β-catenin. At first, we transfected miR-214-3p mimics into cervical cancer cells, mRNA and protein expression of β-catenin were both declined (Fig. 6A, B), indicated that β-catenin indeed was a direct target gene of miR-214-3p. When HOTAIR was knocked down in End1/E6E7 and SiHa, β-catenin mRNA and protein expression level were also significantly inhibited (Fig. 6C, D). But the expression level β-catenin could up-regulate again when HOTAIR-A plasmid contained the binding sites of miR-214-3p was transfected into C-33A cells (Fig. 6E, F). Combining with the previous results, HOTAIR and miR-214-3p could both directly regulated the expression of β-catenin, and HOTAIR could upregulated the expression of β-catenin by targeted adsorbing miR-214-3p in cervical cancer cells. In another word, HOTAIR and β-catenin acted as a pair of competitive endogenous RNA in HPV16 positive cervical cancer through competing binding to miR-214-3p. Finally, Wnt/β-catenin dependent pathway might activate by HOTAIR/ miR-214-3p axis in cervical cancer cells.

## Discussion

Increasing evidence has revealed that specific lncRNAs might give a significant contribution in the progression, metastasis and chemo resistance of cancer [36]. These multiple functional tumor-associated lncRNAs have either oncogenic or tumor-suppressive properties in cancer [37]. In particular, accumulating research data have demonstrated that the lncRNAs alterations in HPV infected cells was also crucial for the progression of cervical cancer. For example, a microarray analysis revealed that thousands of host lncRNAs had differential expression in oncogenic HPV-positive cells compared to the HPV-negative cervical cancer cell line [38]. Lots of lncRNAs were found to be differentially expressed in the HPV18 positive HeLa cells respect to C33A cell line [39]. In addition, accumulating research data have demonstrated the high risk HPV E6 and E7 oncoproteins could alter the expression of these lncRNAs and their downstream miRNAs or targets [38].

In general, HOTAIR has been shown to recruits chromatin-modifying proteins and to affect cancer epigenome modulation [40]. In cervical cancer, limited data suggested that HOTAIR could act as sponge for several miRNAs and cause deregulation of the respective target genes [41, 42]. However, the exact mechanism remain unclear. Furthermore, HOTAIR levels have shown to be strictly controlled by the HPV E7 protein in cervical cancer [24]. Meanwhile, datasets mining also found the higher expression of HOTAIR in cancer tissues [23, 43, 44]. In this study, our results revealed that HOTAIR and miR-214-3p were both differentially expressed in HPV16 positive cervical cancer cells, HOTAIR showed significantly higher expression level, while miR-214-3p remains a lower expression level in cervical cancer cells. In brief, they showed exactly reverse expression tendency.

For better investigate the function of HOTAIR and miR-214-3p in HPV16 positive cervical cancer, we knocked down HOTAIR and upregulated miR-214-3p in HPV16 positive cells, respectively. Our results indicated that no matter knocking down of HOTAIR or overexpressed miR-214-3p, cell proliferate ability was all significantly inhibited accompanied by cellular apoptosis increased. That represents HOTAIR played a promoting role in HPV16 cervical carcinogenesis and the function of miR-214-3p was totally reverse. We supposed that HOTAIR was responsible for miR-214-3p down-regulation in HPV16 positive cervical cancer by serving as a competitive endogenous RNA through sponging mature miR-214-3p in cells. Based on bioinformatics analysis, we identified a potential binding site on HOTAIR transcript against miR-214-3p. Eventually A seven bp seed sequences (CAGCAGG) at 5′ end of miR-214-3p were fully complementary to the 1807–1813 bp on HOTAIR. Moreover, the function of this binding site as a microRNA response elements (MREs)on HOTAIR was also verified by dual luciferase assay. To further confirm the interaction between HOTAIR and miR-214-3p, we detected the change of miR-214-3p expression when HOTAIR expression was interfered in cancer cells. As shown in the results, miR-214-3p expression decreased accompany with the up-regulation of HOTAIR. Our results revealed a strong negative correlation between HOTAIR and miR*-*214-3p expression.

In above results, we had confirmed that HPV16E7 could upregulated HOTAIR expression. Next, when down or up-regulated expression of HPV16 E7 respectively, the expression of HOTAIR showed the same trend. In addition, miR-214-3p expression was significantly increased when HPV16 E7 expression was knocked down, but once HPV16 E7 gene was up-regulated expression again in cancer cells, HOTAIR expression went up also and the expression trend of miR-214-3p was obviously reversed. The results suggest that the infection status of HPV16 played an important role in regulating expression of HOTAIR or miR-214-3p in cervical cancer cells. Consequently, we speculated that the main cause of the above phenomenon was attributed to losing the sponge effect of HOTAIR against miR-214-3p.

Based on bioinformatics analysis, Wnt/β-catenin signaling pathway was one of the key pathways implicated in miR-214-3p regulation network in cervical cancer. Interestingly, Wnt/β-catenin signaling pathway contains some key genes, such as CTNNB1 (β-catenin), PLCB4, PSEN1, PPP2CB, were all the well-recognized target gene for miR-214-3p by bioinformatics analysis. The studies found that miR-214-3p could degrade or inactivate the β-catenin gene by binding to the 3′UTR of β-catenin mRNA in various malignant tumors including breast cancer and esophageal cancer [45, 46]. In our study, β-catenin was significantly decreased at both mRNA and protein level when miR-214-3p was up expressed in HPV16 cervical cancer cells. And once HOTAIR was knocked down in HPV16 positive cervical cancer cells, miR-214-3p could escape from the absorption of HOTAIR and further lead to its target gene β-catenin been degraded. HOTAIR in HPV-negative C-33A cells remains an extremely lower level, so when we overexpressed the HOTAIR functional region in C-33A cells, the free miR-214-3p lost its inhibitory effect against β-catenin mRNA due to the adsorption of HOTAIR against miR-214-3p, eventually caused the expression of β-catenin was significantly up-regulated in cervical cancer cells.

Wnt/β-catenin dependent pathway also known as the canonical pathway. When Wnt/β-catenin dependent pathway was activated, β-catenin protein was accumulated in the cytoplasm, then it was transferred to the nucleus and interacted with T cell factor (TCF)/Lymphoid Enhancer Factor (LEF) transcription factors, and promotes the transcription of downstream targets, such as cyclin D1, c-Myc, and matrix metalloproteinase 1 [47]. On this basis, Wnt/β-catenin signaling pathway played modulation roles in cancer cells apoptosis, proliferation, invasion, and migration in the progression of various cancers [48]. In this study, we found when HOTAIR was knocked down in HPV16 positive cervical cancer cells, the expression of β-catenin was significantly inhibited. We supposed that the lower expression of HOTAIR lost the sponge effect against miR-214-3p, further lead to degradation of β-catenin mRNA, then wnt/β-catenin signaling pathway was inhibited in cells. Oppositely, in HPV16-positive cervical cancer cells, HOTAIR was highly expressed due to the presence of HPV16E7, then miR-214-3p was adsorbed and loses its effect on target genes, and β-catenin expression was released, resulting in malignant phenotype of cervical cancer cells. In summary, HOTAIR and miR-214-3p might form a regulated axis and play an important regulation roles in the malignant behavior of HPV16-positive cervical cancer cells. Also, canonical wnt/β-catenin signaling pathway was a key part of this ceRNA regulatory network in cervical cancer.

Given their stability and distinct cytoplasmic localization, lncRNAs and miRNAs could be used as novel therapeutic target drugs or tools for the treatment of cervical cancers. Our studies clearly highlight the significance of miRNA-214 and HOTAIR signatures in treatment of cervical cancer. For example, the combination of miR-214 overexpression and paclitaxel treatment might be more effective in the treatment of cervical cancer than paclitaxel alone [32]. At present, some RNA-based approaches have been developed to target ncRNAs, including antisense oligonucleotides (ASOs) or siRNAs. These targeting molecules might become effective epigenetic drugs to improve the treatment of cervical cancer [38, 49]. In addition, the other therapeutic agents against HOTAIR such as tumor specific peptides or Dioscin, have been used to inhibit the expression of HOTAIR in ovarian cancer, breast cancer and gastric cancer [50, 51]. Our findings contributed to the development of novel drugs against cervical cancer.

At present, cervical cancer continued to represent an important health problem for in women worldwide. Although the epigenetic alterations in HPV infected cells indeed be crucial for the progression to cervical cancer, studies on the correlation between HPV and intracellular ncRNA were very limited so far. Meanwhile, the different investigations provided conflicting results with a significant proportion of ncRNAs being upregulated in one study but downregulated in another study. Therefore, characterization of the complex relationship between ncRNAs and target genes were needed to further carried out, help to improve the early diagnosis and treatment of cervical cancer.

## Conclusion

In conclusion, we found a novel regulatory axis: HOTAIR/miR-214-3p/β-catenin, exerts a ceRNA effect, involved in proliferation and apoptosis of HPV16 positive cervical cancer cells. Our results provided some potential therapeutic targets and prognostic indicators of cervical cancer.

## Data Availability

All data generated or analyzed during this study are included in this published article.

## Abbreviations

CC: Cervical cancer
HPV: Papillomavirus
QRT-PCR: Quantitative real-time polymerase chain reaction
ceRNA: Competitive endogenous RNA
LncRNA: Long non-coding RNA
miRNA: MicroRNA
UTR: Untranslated region
FBS: Fetal bovine serum
PMSF: Phenylmethanesulfonyl fluoride
